# Efficacy of Duhuo Jisheng Decoction for Treating Cold-Dampness Obstruction Syndrome-Type Knee Osteoarthritis: A Pooled Analysis

**DOI:** 10.1155/2022/2350404

**Published:** 2022-06-21

**Authors:** Jinlong Zhao, Guihong Liang, Jianke Pan, Weiyi Yang, Lingfeng Zeng, Jun Liu

**Affiliations:** ^1^The Second Clinical Medical College of Guangzhou University of Chinese Medicine, Guangzhou 510405, China; ^2^The Research Team on Bone and Joint Degeneration and Injury of Guangdong Provincial Academy of Chinese Medical Sciences, Guangzhou 510120, China; ^3^The Second Affiliated Hospital of Guangzhou University of Chinese Medicine (Guangdong Provincial Hospital of Chinese Medicine), Guangzhou 510120, China; ^4^The Fifth Clinical Medical College of Guangzhou University of Chinese Medicine, Guangzhou 510405, China; ^5^Guangdong Second Traditional Chinese Medicine Hospital (Guangdong Province Enginering Technology Research Institute of Traditional Chinese Medicine), Guangzhou 510095, China

## Abstract

**Aim:**

The aim of this study is to provide evidence of the effect of Duhuo Jisheng decoction (DHJSD) on knee osteoarthritis (KOA) of the cold-dampness obstruction syndrome type.

**Methods:**

We searched PubMed, Embase, the Cochrane Library, the China National Knowledge Infrastructure, the Wanfang database, and the China Biology Medicine for randomized controlled trials (RCTs) evaluating DHJSD or DHJSD combined with other conventional therapies (DHJSD group) compared to conventional therapy (control group) for cold-dampness obstruction syndrome-type KOA. We calculated the pooled odds ratio (OR), mean difference (MD), and 95% confidence interval (CI) using fixed- or random-effects models.

**Results:**

Eleven RCTs, with a total of 895 patients, were included. The results showed that DHJSD could significantly improve the effective rate (OR = 3.13, 95%CI = 2.07 to 4.72, *P* < 0.001), reduce both the WOMAC (MD = −12.06, 95%CI = -16.34 to -7.79, *P* < 0.001) and VAS (MD = −1.02, 95%CI = -1.54 to -0.50, *P* = 0.0001) scores, and reduce the serum IL-6 (MD = −0.80, 95%CI = -0.90 to -0.69, *P* < 0.001) and TNF-*α* (MD = −2.49, 95%CI = -2.77 to -2.21, *P* < 0.001) levels during the treatment of cold-dampness obstruction syndrome-type KOA. The subgroup analysis showed that compared with glucosamine sulfate (GS) alone, DHJSD combined with GS significantly improved the effective rate (OR = 2.59, 95%CI = 1.19 to 5.65, *P* = 0.02) and reduced the WOMAC (MD = −13.83, 95%CI = -16.14 to -11.51, *P* < 0.001) and VAS (MD = −0.91, 95%CI = -1.27 to -0.55, *P* < 0.001) scores. DHJSD + warm-needle acupuncture (WA) lowered the VAS score more than WA alone. There was no significant difference in the decrease in serum IL-1*β* between the DHJSD and control groups.

**Conclusion:**

This study shows that DHJSD can improve the clinical efficacy and reduce the VAS and WOMAC scores in the treatment of cold-dampness obstruction syndrome-type KOA. Compared with GS or WA alone, the combined application of DHJSD with GS or WA could better reduce both the VAS and WOMAC scores.

## 1. Introduction

Knee osteoarthritis (KOA) is a common disease characterized by progressive articular cartilage degeneration, and its main clinical manifestations are joint pain and dysfunction [1, 2]. As the age of the world population and number of obese people have increased, the global prevalence of KOA has shown a significant increasing trend [3, 4]. KOA not only increases the health burden to individuals and families but also negatively affects the national health-care system and increases socioeconomic costs [5]. Currently, the consensus regarding the KOA ladder treatment scheme is that treatment for early-stage KOA should comprise basic therapy and drug treatment, while the treatments for the middle and late stages should be based on restorative and reconstructive treatments. In terms of drug treatment, nonsteroidal anti-inflammatory drugs are widely used and recognized, but they have the disadvantages of numerous side effects, such as constipation and gastrointestinal discomfort [3, 6]. Therefore, it is particularly important to identify safer and more effective treatments.

KOA belongs to the category of “*Bi Zheng*” in traditional Chinese medicine (TCM), and its pathogenesis is more common in states of external cold and dampness and in deficiencies of the liver and kidney. TCM emphasizes the importance of the accurate identification of disease syndrome types, which is conducive to the accurate usage of TCM to treat diseases. The results of a study involving the distribution of TCM syndrome types among 28,763 patients with KOA showed that cold-dampness obstruction syndrome (21.33%) was the main syndrome type of KOA [7]. Duhuo Jisheng decoction (DHJSD) is the main prescription for the treatment of cold-dampness obstruction syndrome-type KOA, and the main reason is that the herbs included in DHJSD can specifically treat the pathogenic factors of cold-dampness obstruction syndrome-type KOA, such as kidney deficiency, *coldness*, and *dampness* [8]. Previous studies have shown that DHJSD may have potential clinical benefits in the treatment of KOA [9, 10], but further research is still needed. The results of a previous study show that DHJSD can downregulate the expression of TNF-*α*, IL-6, MMP-1, MMP-9, MMP-13, and ADAMTS-5; inhibit the NF-*κ*B and p38 MAPK signaling pathways; activate the AMPK-SIRT1 signaling pathway; inhibit chondrocyte apoptosis; and then play a role in the treatment of osteoarthritis [11]. The previous studies did not distinguish the specific TCM syndrome types that were present in their KOA cases [9, 10], and they used DHJSD for all of their patients, which is not in line with the syndrome differentiation and treatment principles of TCM. In line with precision medicine, clarifying the clinical efficacy of DHJSD in the treatment of KOA by classifying the specific syndrome types (cold-dampness obstruction syndrome) is conducive to promoting the precise application and use of DHJSD in the clinic. Therefore, this study systematically evaluated the efficacy of DHJSD in the treatment of cold-dampness obstruction syndrome-type KOA by meta-analysis to provide evidence-based medical information for its clinical application.

## 2. Materials and Methods

### 2.1. Data Sources

The data sources included PubMed, Embase, the Cochrane Library, the China National Knowledge Infrastructure (CNKI), the Wanfang database, and the China Biology Medicine (CBM).

### 2.2. Search Strategy

The search terms included Duhuo Jisheng, Duhuo Jisheng decoction, Duhuo Jisheng Tang, Duhuo Jisheng, knee osteoarthritis, knee osteoarthritis, osteoarthritis, and knee arthritis. We used the combination of subject words and free words to construct the retrieval model. The retrieval time limit was defined from the establishment of the database until March 12, 2022. The retrieval strategy of each database is shown in supplementary material 1.

### 2.3. Inclusion and Exclusion Criteria

The inclusion criteria were as follows: (1) randomized controlled trials (RCTs), regardless of whether a blinding method was used; (2) studies written in either Chinese or English; (3) reports with clear descriptions of the diagnostic criteria of KOA in patients and differentiation of patients with cold-dampness obstruction syndrome according to the diagnostic criteria of the TCM syndrome type; and (4) intervention measures wherein the control group was treated with conventional Western medicine or a non-TCM decoction (control group) and the treatment group was treated with DHJSD alone or DHJSD combined with conventional Western medicine based on the treatment given to the control group (DHJSD group). The exclusion criteria were (1) reviews, animal experiments, and case reports; (2) duplicate publications; and (3) studies in which valid data could not be extracted.

### 2.4. Outcome Indicators

At least one of the following outcome indicators had to be reported in the included literature: (1) the total effective rate; (2) the total Western Ontario and McMaster University Osteoarthritis Index Scale (WOMAC) score, which is used to evaluate the severity of arthritis and its therapeutic effect according to relevant patient symptoms and signs and the structure and function of the knee joint according to three aspects, i.e., pain, stiffness, and joint function, with a lower WOMAC score indicating a better curative effect; (3) the visual analogue scale (VAS) score, which is used to evaluate the degree of pain from 0 (no pain) to 10 (worst pain); (4) the serum IL-6 level; (5) the serum IL-1*β* level; and (6) the serum TNF-*α* level.

### 2.5. Data Extraction and Quality Evaluation

Two researchers independently completed the literature screening, data extraction, and cross-check. The data extracted included the basic information of the study (author, year of publication, sample size, intervention measures, etc.), the elements for evaluating the risk of bias, and the outcome index data. The bias risk assessment tool recommended in the Cochrane evaluation manual 5.1 was used to evaluate the quality of the included studies.

### 2.6. Statistical Analysis

Rev Man 5.3 was used to process and analyze the extracted data. *P* < 0.05 indicates that the difference is statistically significant. Heterogeneity was evaluated by means of the chi-squared test, and the degree of heterogeneity was measured by *I*^2^ statistics. For the meta-analysis, the fixed-effects model was used when *P* > 0.05 and *I*^2^ < 50%, and the random-effects model was used when *P* < 0.05 or *I*^2^ > 50%. The mean difference (MD) and odds ratio (OR) were used as the effect indexes for the measurement and counting data, and the 95% confidence intervals (CIs) of each effect were calculated.

## 3. Results

### 3.1. Literature Search Results

**Figure 1:**
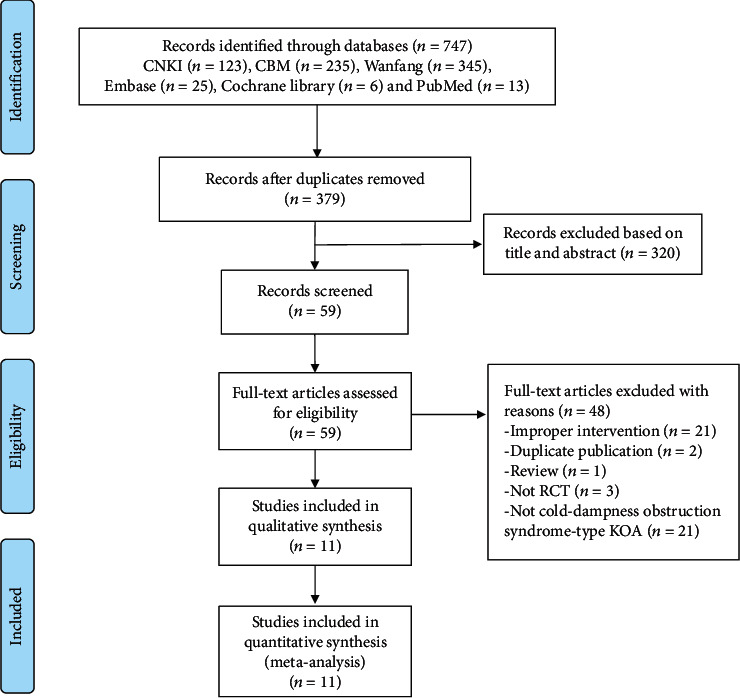
PRISMA flowchart.

We preliminarily retrieved 747 studies, including 13 in PubMed, 25 in Embase, 6 in the Cochrane Library, 123 in the CNKI, 235 in CBM, and 345 in the Wanfang database. After eliminating the duplicate literature, a total of 379 articles were included. A total of 320 studies were eliminated after reading the titles and abstracts. Finally, 11 RCTs were included [12–22]. The process and results of the literature screening are shown in Figure 1.

### 3.2. Basic Information of the Included Studies

A total of 895 patients from 11 RCTs [12–22] were included, and there were 428 patients in the DHJSD group and 467 patients in the control group. Eight studies had a course of treatment and follow-up time of 4 weeks [13, 15–20, 22]. The basic characteristics of the included literature are shown in Table 1. The herbal medicine compositions and dosages of DHJSD that were used in the studies are shown in supplementary material 2.

### 3.3. Literature Quality Evaluation

**Figure 2:**
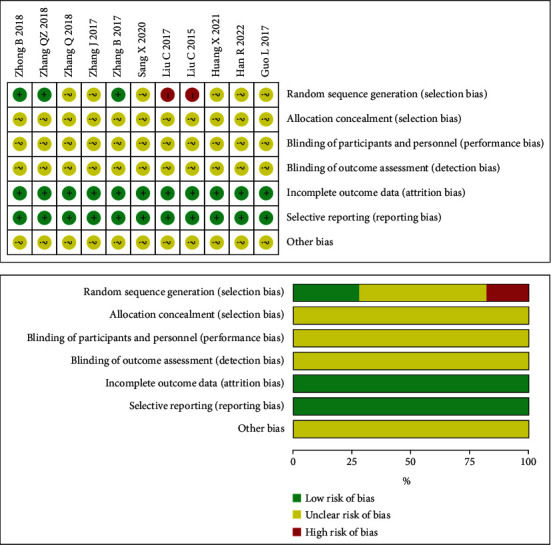
Risk of bias.

The results of the bias risk assessment of the included literature are shown in Figure 2. Two studies were judged as high risk because they did not use the random allocation method [19, 21]. The allocation concealment and scheme implementer blinding methods showed unclear risks. All 11 studies had complete research data. Additionally, we had no evidence to believe that these 11 studies had selective reporting, so these two evaluation indicators were determined to be low risk. The risk of other biases is unclear. Overall, except for the shortcomings of the blind method setting and random distribution concealment, the risk of bias in other aspects is less likely. Therefore, the quality of the literature included in this study is acceptable.

### 3.4. Meta-analysis Results

#### 3.4.1. Efficiency

**Figure 3:**
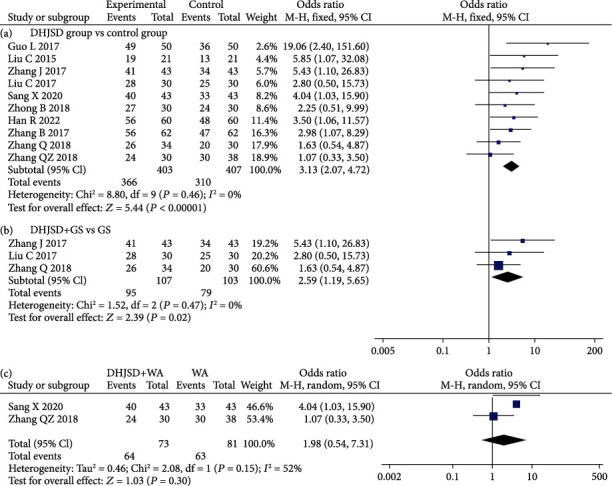
Forest plot for efficacy rate. (a) DHJSD group vs. control group, (b) DHJSD+GS vs. GS, and (c) DHJSD+WA vs. WA.

A total of 10 studies reported the effective rates [12, 14–22]. There were 403 patients in the DHJSD group and 407 patients in the control group. There was no heterogeneity among the studies (*I*^2^ = 0%), so the fixed-effects model was used. The meta-analysis showed that the total effective rate of the DHJSD group was higher than that of the control group (OR = 3.13, 95%CI = 2.07 to 4.72), and the difference was statistically significant (*P* < 0.001). A subgroup analysis showed that the total effective rate of DHJSD + GS was higher than that of GS alone (OR = 2.59, 95%CI = 1.19 to 5.65), and the difference was statistically significant (*P* = 0.02). There was no significant difference in the efficiency between DHJSD + WA and WA (OR = 1.98, 95%CI = 0.54 to 7.31, *P* = 0.30) (Figure 3).

#### 3.4.2. WOMAC Score

**Figure 4:**
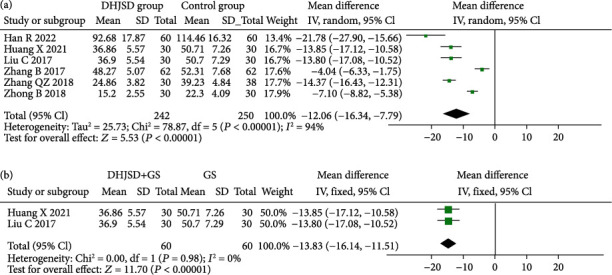
Forest plot for WOMAC score. (a) DHJSD group vs. control group and (b) DHJSD+GS vs. GS.

A total of six studies reported WOMAC scores [12, 13, 15, 17, 19, 22]. There were 492 patients, including 242 in the DHJSD group and 250 in the control group. The heterogeneity test showed that there was heterogeneity between the two groups (*I*^2^ = 94%), so the random-effects model was used. The results of the meta-analysis showed that the total WOMAC score in the DHJSD group was lower than that in the control group (MD = −12.06, 95%CI = -16.34 to -7.79), and the difference was statistically significant (*P* < 0.001). The subgroup analysis showed that DHJSD + GS could reduce WOMAC scores more than GS alone (MD = −13.83, 95%CI = -16.14 to -11.51), and the difference was statistically significant (*P* < 0.001) (Figure 4).

#### 3.4.3. VAS Score

**Figure 5:**
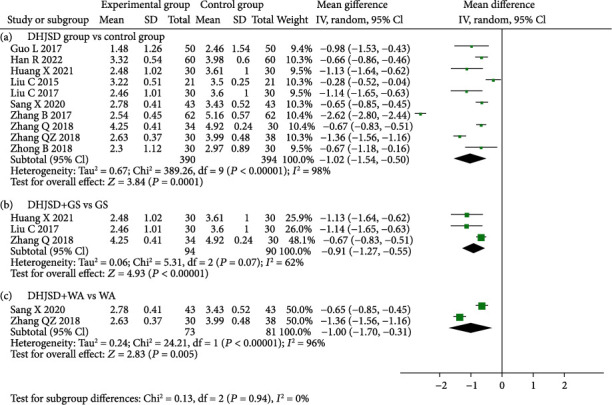
Forest plot for VAS score. (a) DHJSD group vs. control group, (b) DHJSD+GS vs. GS, and (c) DHJSD+WA vs. WA.

A total of 10 studies reported VAS scores [12–17, 19–22]. There was heterogeneity among the various studies (*I*^2^ = 98%), so the random-effects model was adopted. The results of the meta-analysis showed that DHJSD could reduce the VAS score more than the treatments used in the control group (MD = −1.02, 95%CI = -1.54 to -0.50), and the difference was statistically significant (*P* = 0.0001). The subgroup analysis showed that DHJSD + GS could reduce the VAS score more than GS alone (MD = −0.91, 95%CI = -1.27 to -0.55), and the difference was statistically significant (*P* < 0.001). DHJSD + WA reduced the VAS score more than WA (MD = −1.00, 95%CI = -1.70 to -0.31, *P* = 0.005) (Figure 5).

#### 3.4.4. Serum IL-6, IL-1*β*, and TNF-*α* Levels

**Figure 6:**
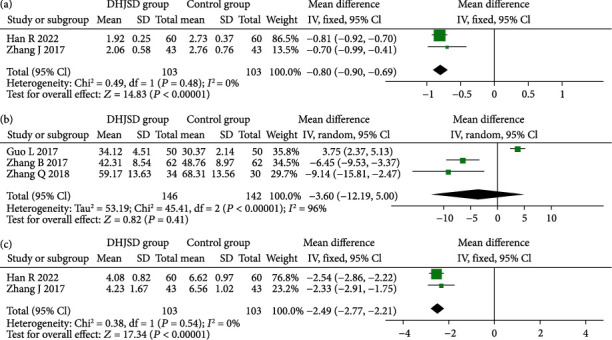
Forest plot of DHJSD group vs. control group on changing (a) IL-6, (b) IL-1*β*, and (c) TNF-*α*.

We conducted a meta-analysis of three objective inflammatory indicators. The results showed that the DHJSD group had a higher reduction in serum IL-6 (MD = −0.80, 95%CI = -0.90 to -0.69, *P* < 0.001) and TNF-*α* (MD = −2.49, 95%CI = -2.77 to -2.21, *P* < 0.001) than the control group. There was no significant difference in the reduction in serum IL-1*β* between the DHJSD group and the control group (MD = −3.60, 95%CI = -11.19 to 5.00, *P* = 0.41) (Figure 6).

### 3.5. Publication Bias Analysis

**Figure 7:**
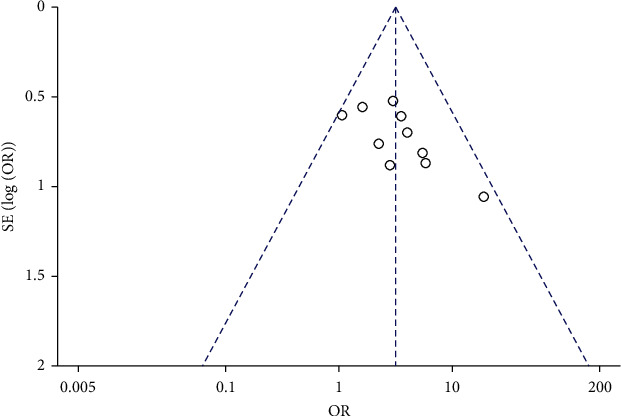
Funnel plot of the total effective rate.

An inverted funnel chart was drawn with the effective rate of 10 included studies as the index. The scattered points in the funnel diagram are basically symmetrical, suggesting that there is no evidence of publication bias in the evaluation of the effective rate of DHJSD for the treatment of cold-dampness obstruction syndrome-type KOA (Figure 7).

## 4. Discussion

The treatment methods for KOA include mainly surgical treatment, drug treatment, and physical therapy [23–25], and drug therapy for KOA comprises mainly nonsteroidal anti-inflammatory drugs and glucosamine [26]. Although the application of these drugs alleviates the pain or improves the activity of KOA patients, the objective adverse reactions and high costs of these drugs are still key problems that need to be solved. TCM emphasizes syndrome differentiation and treatment, which is the application of precision medicine. DHJSD is used mainly to treat cold-dampness obstruction syndrome-type KOA, but there is still a lack of advanced evidence to prove this view.

For the first time, this study systematically evaluated the intervention effect of DHJSD in the treatment of KOA with cold-dampness obstruction. The results of this meta-analysis show that DHJSD alone or combined with other conventional therapies can significantly improve the effective rate in the treatment of cold-dampness obstruction syndrome-type KOA, reduce the WOMAC and VAS scores, and reduce serum IL-6 and TNF-*α* levels. The subgroup analysis showed that compared with GS alone, DHJSD combined with GS could significantly improve the effective rate and reduce both the WOMAC score and VAS score, which showed that the combined application of DHJSD and GS was worthy of clinical application. DHJSD + WA can lower VAS scores more than WA alone, which suggests that the effect of DHJSD combined with WA may be better in patients with symptoms of pain.

The results of a prospective clinical study showed that the use of DHJSD for 4 weeks in the treatment of KOA can alleviate knee pain and stiffness [9], which is consistent with our research conclusions. An experimental study previously confirmed that DHJSD can bypass the TLR4/MyD88/NF-*κ*B signaling pathway and that DHJSD plays an anti-inflammatory role, which is considered to be the pharmacological mechanism of DHJSD in the treatment of OA [27]. A study also suggested that DHJSD can promote G1/S checkpoint conversion in the cell cycle; upregulate the protein expression of D1, CDK4, Cdk6, and Rb; downregulate p16; and promote chondrocyte proliferation [28]. DHJSD inhibits tunicamycin-induced endoplasmic reticulum stress by downregulating miR-34a, suggesting that DHJSD may be a potential treatment for KOA [29]. A previous clinical study also showed that DHJSD has satisfactory efficacy in improving pain and activity in patients with KOA [9]. Combined with the results of this study, we believe that DHJSD has potential therapeutic advantages in the treatment of cold-dampness obstruction-type KOA; therefore, the accurate application of this TCM formula can be used for the treatment of this type of KOA. In addition, the results of this study showed that DHJSD did not have a better effect on reducing serum levels of IL-6 and TNF-*α*. On the basis of the application guidance and our expertise in TCM, we make the following two assumptions. The treatment course included in the study was mainly 4 weeks, which led us to speculate that the treatment effect against inflammatory factors may not be completely due to the short treatment course. Second, since the last follow-up time included in the study is basically at the end of the course of treatment, it may not provide enough time to observe the intervention effect of DHJSD on inflammatory factors. Therefore, future research is needed to further explore the role of different courses of DHJSD in the treatment of KOA, and long-term clinical follow-up is necessary.

This study has the following limitations: (1) The severities of the included subjects' KOA were not completely consistent, which may affect the reliability of the results obtained after evaluating the outcome indicators. (2) Due to the mixed factors affecting the occurrence and development of KOA, we did not include descriptions and matching tests for this type of information. (3) The included literature of this study describes the diagnostic criteria of cold-dampness obstruction syndrome; however, because each study does not adopt the same syndrome-type diagnostic method, the reliability of the syndrome-type diagnosis may be affected. Therefore, we suggest that future research use the unified diagnostic criteria of TCM syndrome types under the current standards. (4) Additionally, the included literature was low quality, suggesting that the future direction should be to include more high-quality clinical RCTs to further verify the conclusions of this study.

## 5. Conclusion

The results of this study show that DHJSD can improve the clinical effective rate and can reduce both the VAS and WOMAC scores in the treatment of cold-dampness obstruction syndrome-type KOA. The subgroup analysis showed that on the basis of GS or WA alone, the combined application of DHJSD could better reduce the VAS and WOMAC scores. The conclusions of this study need to be confirmed by more reasonably designed, high-quality, large-sample, multicenter, randomized double-blind controlled clinical trials.

## Figures and Tables

**Table 1:** Characteristics of the included randomized controlled trials.

First author	Year	Interventions		Sample size (male/female)		Age (years, mean)		Duration in years		Duration of treatment	Follow-up
		DHJSD	Control	DHJSD	Control	DHJSD	Control	DHJSD	Control		
Han R [12]	2022	DHJSD+C	PRP	60 (38/22)	60 (37/23)	65.29 ± 2.17	65.28 ± 2.15	5.77 ± 1.61	5.72 ± 1.62	3 weeks	3 weeks
Huang X [13]	2021	DHJSD	GS	30 (-/-)	30 (−/−)∗	Overall: 45 to 75		—	—	4 weeks	Not stated
Sang X [14]	2020	DHJSD+C	WA	43 (18/25)	43 (17/26)	59.85 ± 10.3	60.34 ± 9.86	5.63 ± 2.58	5.78 ± 3.15	3 weeks	3 weeks
Zhong B [15]	2018	DHJSD+C	Intradermal needling	30 (17/13)	30 (21/9)	46.9 ± 8.11	47.7 ± 9.44	—	—	8 weeks	8 weeks
Zhang Q [16]	2018	DHJSD	GS	34 (18/16)	30 (14/16)	—	—	—	—	4 weeks	4 weeks
Zhang B [17]	2017	DHJSD+C	Diclofenac sodium	62 (36/26)	62 (37/25)	62.13 ± 6.54	62.24 ± 6.49	13.27 ± 6.56^▲^	12.34 ± 6.47^▲^	4 weeks	4 weeks
Zhang J [18]	2017	DHJSD	GS	43 (18/25)	43 (17/26)	71 ± 5.27	69 ± 6.58	5.1 ± 1.5	5.5 ± 0.5	4 weeks	4 weeks
Liu C [19]	2017	DHJSD	GS	30 (12/18)	30 (13/17)	62.67	62.53	—	—	4 weeks	4 weeks
Guo L [20]	2017	DHJSD	Cataplasm	50 (27/23)	50 (28/22)	59.78 ± 1.37	59.84 ± 1.62	7.36 ± 0.84	7.24 ± 0.76	4 weeks	4 weeks
Liu C [21]	2015	DHJSD	Ibuprofen	21 (14/7)	21 (10/11)	61.52 ± 2.68	62.37 ± 3.02	4.69 ± 2.55^▲^	4.71 ± 1.92^▲^	2 months	2 months
Zhang QZ [22]	2018	DHJSD+C	WA	30 (16/14)	38 (18/20)	56.44 ± 7.07	55.93 ± 7.46	—	—	4 weeks	4 weeks

DHJSD: Duhuo Jisheng decoction; WA: warm-needle acupuncture; GS: glucosamine sulfate; PRP: platelet-rich plasma. ∗There were 28 males and 32 females. ▲: months.

## Data Availability

The dataset can be accessed from the corresponding author upon reasonable request.

## Supplementary Materials

Supplementary Materials S1: searching strategies for all databases. S2: Composition of formula in each included randomized controlled trials.
