# Supplementary material for: Efficacy of Duhuo Jisheng Decoction for Treating Cold-Dampness Obstruction Syndrome-Type Knee Osteoarthritis: A Pooled Analysis
Source: Biomed Res Int. 2022 Jun 21;2022:2350404. doi: 10.1155/2022/2350404 (PMC9239816; doi:10.1155/2022/2350404)
Supplement: Supplementary Materials — S1: searching strategies for all databases. S2: Composition of formula in each included randomized controlled trials. [file 2350404.f1.zip › S1 Searching strategies for all databases.docx]

**S1: Searching strategies for all databases.**

**CNKI**

TKA = 'Duhuo Jisheng Decoction' AND (TKA = 'knee osteoarthritis' OR TKA = 'osteoarthritis' OR TKA = 'knee arthritis' OR TKA = 'osteoarthritis, knee') AND TKA = 'dampness'

**Wanfang**

Title or Key words: ("knee osteoarthritis" or "osteoarthritis" or "knee arthritis" or "osteoarthritis, knee") and Title or Key words: (Duhuo Jisheng Decoction) and Abstract: (dampness)

**CBM**

#1 "knee osteoarthritis"[Common fields] OR "osteoarthritis"[Common fields] OR "knee arthritis"[Common fields] OR "osteoarthritis, knee"[Common fields]

#2 "Duhuo Jisheng Decoction"[Title]

#3 "dampness"[Abstract]

#4 (#3) AND (#2) AND (#1)

**Embase**

#1 'knee osteoarthritis':ti,ab,kw OR osteoarthritis:ti,ab,kw OR 'knee arthritis':ti,ab,kw

#2 'duhuo jisheng':ti,ab,kw OR 'duhuo jisheng decoction':ti,ab,kw OR 'duhuo jisheng tang':ti,ab,kw OR duhuojisheng:ti,ab,kw

#3 #1 AND #2

**PubMed**

#1 ((((Duhuo Jisheng[Title/Abstract]) OR (Duhuo Jisheng Decoction[Title/Abstract])) OR (Duhuo Jisheng Tang[Title/Abstract])) OR (DuhuoJisheng[Title/Abstract])) AND

#2 (((((knee osteoarthritis[Title/Abstract]) OR (Knee Osteoarthritis[Title/Abstract])) OR (Osteoarthritis[Title/Abstract])) OR (knee arthritis[Title/Abstract])) OR (osteoarthritis, knee[MeSH Terms]))

#3 #1 AND #2

**Cochrane Library**

#1 (Duhuo Jisheng):ti,ab,kw OR (Duhuo Jisheng Decoction):ti,ab,kw OR (Duhuo Jisheng Tang):ti,ab,kw OR (DuhuoJisheng):ti,ab,kw

#2 (knee osteoarthritis):ti,ab,kw OR ("knee osteoarthritis"):ti,ab,kw OR (Osteoarthritis):ti,ab,kw OR (knee arthritis):ti,ab,kw

#3 #1 AND #2
