# Supplementary material for: Efficacy of Duhuo Jisheng Decoction for Treating Cold-Dampness Obstruction Syndrome-Type Knee Osteoarthritis: A Pooled Analysis
Source: Biomed Res Int. 2022 Jun 21;2022:2350404. doi: 10.1155/2022/2350404 (PMC9239816; doi:10.1155/2022/2350404)
Supplement: Supplementary Materials — S1: searching strategies for all databases. S2: Composition of formula in each included randomized controlled trials. [file 2350404.f1.zip › S2 Composition of formula.docx]

S2 Composition of formula in each included randomized controlled trials.

| Study ID | Composition of formula. |
| --- | --- |
| Huang X[10] | Not report. |
| Zhang B [14] | Radix Angelicae Pubescentis (Du-huo), Herba Taxilli (Sang-ji-sheng), Cortex Eucommiae (Du-zhong), Radix Achyranthis Bidentatae (Niu-xi), Erba Asari (Xi-xin), Radix Gentianae Macrophyllae (Qin-jiao), Poria (Fu-ling), Cortex Cinnamomi (Rou-gui), Radix Ledebouriellae (Fang-feng), Rhizoma Chuanxiong (Chuan-xiong), Aralia ginseng (Ren-shen), Radix Glycyrrhizae (Gan-cao), Radix Angelicae Sinensis (Danggui), Paeonia lactiflora Pall (Shao-yao), Rehmannia (Di-huang) |
| Zhang J [15] | Radix Angelicae Pubescentis (Du-huo) 15g, Cortex Eucommiae (Du-zhong) 15g, Radix Achyranthis Bidentatae (Niu-xi) 15g, Poria (Fu-ling) 15g, Radix Angelicae Sinensis (Dang gui) 15g, Radix Paeoniae Alba (Bai-shao) 15g, Radix Rehmanniae Preparata (Shu-di-huang) 15g, Radix Codonopsis (Dang-shen) 20g, Herba Taxilli (Sang-ji-sheng) 20g, Erba Asari (Xi-xin) 6g, Cortex Cinnamomi (Rou-gui) 6g, Radix Glycyrrhizae (Gan-cao) 6g, Radix Gentianae Macrophyllae (Qin-jiao) 10g, Radix Ledebouriellae (Fang-feng) 10g, Rhizoma Chuanxiong (Chuan-xiong) 10g |
| Zhong B [12] | Radix Angelicae Pubescentis (Du-huo) 10g, Herba Taxilli (Sang-ji-sheng) 12g, Cortex Eucommiae (Du-zhong) 15g, Radix Achyranthis Bidentatae (Niu-xi) 10g, Erba Asari (Xi-xin) 3g, Radix Gentianae Macrophyllae (Qin-jiao) 10g, Fu-Cha 10g, Cortex Cinnamomi (Rou-gui) 6g, Radix Ledebouriellae (Fang-feng) 10g, Rhizoma Chuanxiong (Chuan-xiong) 10g, Radix Codonopsis (Dang-shen) 15g, Radix Glycyrrhizae (Gan-cao) 5g, Radix Angelicae Sinensis (Danggui) 10g, Radix Paeoniae Alba (Bai-shao) 15g, Radix Rehmanniae Preparata (Shu-di-huang) 15g |
| Liu C [16] | Radix Angelicae Pubescentis (Du-huo) 9g, Radix Achyranthis Bidentatae (Niu-xi) 6g, Herba Taxilli (Sang-ji-sheng) 6g, Cortex Eucommiae (Du-zhong) 6g, Radix Gentianae Macrophyllae (Qin-jiao) 6g, Cortex Cinnamomi (Rou-gui) 6g, Erba Asari (Xi-xin) 3g, Poria (Fu-ling) 6g, Radix Ledebouriellae (Fang-feng) 9g, Radix Angelicae Sinensis (Danggui) 6g, Aralia ginseng (Ren-shen) 6g, Rhizoma Chuanxiong (Chuan-xiong) 6g, Radix Glycyrrhizae (Gan-cao) 6g, Paeonia lactiflora Pall (Shao-yao) 6g, Astragalus membranaceus (Huang-qi) 10g, Dipsacales (Xu-duan) 10g, Rehmannia (Di-huang) 6g, Psoralea corylifolia (Bu-guzhi) 10g, OsDraconis (Long-gu) 10g, ostrea gigas thunberg (Mu-li) 10g |
| Han R [9] | Radix Codonopsis (Dang-shen) 20g, Herba Taxilli (Sang-ji-sheng) 20ｇ, Radix Achyranthis Bidentatae (Niu-xi) 15g, Cortex Eucommiae (Du-zhong) 15g, Radix Rehmanniae Preparata (Shu-di-huang) 15g, Poria (Fu-ling) 15g, Radix Angelicae Sinensis (Danggui) 15g, Radix Angelicae Pubescentis (Du-huo) 15g, Radix Paeoniae Alba (Bai-shao) 15g, Radix Gentianae Macrophyllae (Qin-jiao) 10g, Radix Ledebouriellae (Fang-feng) 10g, Rhizoma Chuanxiong (Chuan-xiong) 10g, Radix Glycyrrhizae (Gan-cao) 6g, Erba Asari (Xi-xin) 6g, Cortex Cinnamomi (Rou-gui)6g |
| Zhang Q[13] | Radix Angelicae Pubescentis (Du-huo) 9g, Herba Taxilli (Sang-ji-sheng) 6g, Radix Angelicae Sinensis (Danggui) 6g, Poria (Fu-ling) 6g, Radix Codonopsis (Dang-shen) 6g, Radix Rehmanniae Preparata (Shu-di-huang) 6g, Radix Ledebouriellae (Fang-feng)6g, Radix Gentianae Macrophyllae (Qin-jiao) 6g, Radix Paeoniae Alba (Bai-shao) 6g, Radix Achyranthis Bidentatae (Niu-xi) 6g, Rhizoma Chuanxiong (Chuan-xiong)  6g, Radix Aconiti (Chuan-wu) 6g, Erba Asari (Xi-xin) 3g, Radix Glycyrrhizae (Gan-cao) 6g |
| Guo L [17] | Radix Glycyrrhizae (Gan-cao) 9g, Radix Angelicae Pubescentis (Du-huo) 9g, Radix Ledebouriellae (Fang-feng) 6g, Radix Angelicae Sinensis (Danggui) 6g, Cortex Eucommiae (Du-zhong) 6g, Radix Codonopsis (Dang-shen) 6g, Radix Achyranthis Bidentatae (Niu-xi) 6g, Paeonia lactiflora Pall (Shao-yao) 6g, Herba Taxilli (Sang-ji-sheng) 6g, Radix Gentianae Macrophyllae (Qin-jiao) 6g, Rehmannia (Di-huang) 6g, Poria (Fu-ling) 6g, Rhizoma Chuanxiong (Chuan-xiong) 6g, Erba Asari (Xi-xin) 3g, Cortex Cinnamomi (Rou-gui) 3g |
| Liu C [18] | Rhizoma Chuanxiong (Chuan-xiong) 30g, Radix Angelicae Pubescentis (Du-huo) 15g, Poria (Fu-ling) 15g, Radix Gentianae Macrophyllae (Qin-jiao) 15g, Radix Codonopsis (Dang-shen) 15g, Radix Angelicae Sinensis (Danggui) 12g, Herba Taxilli (Sang-ji-sheng) 10g, Cortex Eucommiae (Du-zhong) 10g, Radix Ledebouriellae (Fang-feng) 10g, Radix Achyranthis Bidentatae (Niu-xi) 10g, Rehmannia (Di-huang) 9g, Paeonia lactiflora Pall (Shao-yao) 6g, Radix Glycyrrhizae (Gan-cao) 6g, Cortex Cinnamomi (Rou-gui) 6g, Erba Asari (Xi-xin) 6g |
| Sang X [11] | Radix Angelicae Pubescentis (Du-huo) 10g, Herba Taxilli (Sang-ji-sheng) 10g, Radix Angelicae Sinensis (Danggui) 9g, Cortex Eucommiae (Du-zhong) 9g, Radix Ledebouriellae (Fang-feng) 9g, Poria (Fu-ling) 9g, Radix Achyranthis Bidentatae (Niu-xi) 9g, Rhizoma Chuanxiong (Chuan-xiong) 6g, Radix Paeoniae Alba (Bai-shao) 6g, Erba Asari (Xi-xin) 6g, Radix Gentianae Macrophyllae (Qin-jiao) 6g, Radix Codonopsis (Dang-shen) 6g, Radix Rehmanniae Preparata (Shu-di-huang) 6g, Radix Glycyrrhizae (Gan-cao) 6g |
| Zhang QZ [19] | Radix Angelicae Pubescentis (Du-huo) 9g, Herba Taxilli (Sang-ji-sheng) 6g, Radix Angelicae Sinensis (Danggui) 6g, Poria (Fu-ling) 6g, Radix Codonopsis (Dang-shen) 6g, Radix Rehmanniae Preparata (Shu-di-huang) 6g, Radix Ledebouriellae (Fang-feng) 6g, Radix Gentianae Macrophyllae (Qin-jiao) 6g, Radix Paeoniae Alba (Bai-shao)6g, Radix Achyranthis Bidentatae (Niu-xi) 6g, Rhizoma Chuanxiong (Chuan-xiong) 6g, Radix Aconiti (Chuan-wu) 6g, Erba Asari (Xi-xin) 6g, Radix Glycyrrhizae (Gan-cao) 6g |

**References:**

[9] R. Han, "Clinical Observation of Duhuo Jisheng Deconction Combined with Autologous Platelet-rich Plasma in the Treatment of Knee Osteoarthritis of Win Cold Dampness Type," *Asia-Pacific Traditional Medicine*, vol. 18, no. 01, pp. 109-112, 2022.

[10] X. Huang, J. Yao, Y. Cai, F. Liu, X. Li, "Clinical observation on treating knee osteoarthritis ofwind-cold-dampness type with Duhuojisheng Decoction," *Osteoarthritis AND Cartilage*, vol. 29, pp. S232, 2021.

[11] X. Sang, "Effectiveness Evaluation on Needle Warming Moxibustion Combined with Duhuo Jisheng Decoction in Treating Wind-Cold-Dampness KOA," *Reflexology and Rehabilitation Medicine*, vol. 29, no. 11, pp. 55-57, 2020.

[12] B. Zhong, "Clinical observation on the treatment of cold-damp obstruction type of knee Osteoarthritis with Duhuojisheng Decoction Combine with needle-embedding," *Nanjing University of Chinese Medicine*, 2018.

[13] Q. Zhang, Y. Miao, D. L Wang, S. Q. Chen, H. N. Li, "Efficacy of Duhuo Jisheng Tang Combined with Warm Acupuncture in Treating Patients with Wind-cold Dampness-type Knee Osteoarthritis and Effect on Serum Pain Mediators，Inflammatory Mediators and Leukotrienes," *Chinese Journal of Experimental Traditional Medical Formulae*, vol. 24, no. 18, pp. 153-158, 2018.

[14] B. S. Zhang, "Effect of Duhuo Jisheng Tang on Synovial Fluid Inflammatory Factors, Oxidative Stress and Adipokinesin Patients with Wind-cold-damp Retention Type Arthritis," *Chinese Journal of Experimental Traditional Medical Formulae*, vol. 23, no. 06, pp. 186-191, 2017.

[15] J. Zhang, Z. J. Liang, Z. B. Huang, R. S. Lin, "Effect of Duhuojisheng Decoction on knee osteoarthritis of rheumatism and cold arthralgia type and its effect on inflammatory factors," *Shaanxi Journal of Traditional Chinese Medicine*, vol. 38, no. 09, pp. 1226-1228, 2017.

[16] C. Liu, "CLINICAL OBSERVATION OF DAMPNESS TYPE OF KNEE OSTEOARTHRITIS TREATED BY DUHUO JISHENG DECOCTION,"*Anhui University of Chinese Medicine* , 2017.

[17] L, Guo, "Clinical analysis of Duhuojisheng Decoction in the treatment of knee osteoarthritis of wind cold dampness arthralgia type," *J Medical Forum*, vol. 38, no. 01, pp. 139-140, 2017.

[18] C. Liu, "Blockage Alpine Type of Knee Osteoarthritis Randomized Parallel Group Study Duhuo Jisheng Soup Treated," *Journal of Practical Traditional Chinese Internal Medicine*, vol. 29, no. 05, pp. 45-47, 2015.

[19] Q. Z. Zhang, Y. Miao, D. L. Wang, J. Li, S. Q. Chen, H. N. Li, "Clinical Ｒandomized Controlled Study of Warming Acupuncture Combined with Duhuo Jisheng Decoction in Treatment of Patients with Wind - cold Dampness - type Knee Osteoarthritis and Changes of ASF1a and SIＲT1 in Joint Fluid," *Liaoning Journal of Traditional Chinese Medicine*, vol. 45, no. 12, pp. 2624-2628, 2018.
